# Mechanical wear analysis helps understand a mechanism of failure in retrieved magnetically controlled growing rods: a retrieval study

**DOI:** 10.1186/s12891-020-03543-4

**Published:** 2020-08-05

**Authors:** Jack Z. Wei, Harry S. Hothi, Holly Morganti, Sean Bergiers, Elisabetta Dal Gal, Doris Likcani, Johann Henckel, Alister J. Hart

**Affiliations:** 1 Institute of Orthopaedics and Musculoskeletal Science, University College London, Brockley Hill, Stanmore, HA7 4LP UK; 2 Royal National Orthopaedic Hospital, Brockley Hill, Stanmore, HA7 4LP UK

**Keywords:** Early-onset scoliosis, Magnetic expansion control growing rods, Internal mechanism, Mechanical wear, Material loss

## Abstract

**Background:**

To assess the relationship between mechanical wear and the failure of the internal lengthening mechanism in retrieved MAGnetic Expansion Control (MAGEC) growing rods.

**Methods:**

This study included 34 MAGEC rods retrieved from 20 patients. The state of the internal mechanism and mechanical wear were assessed in all the rods using plain radiographs and visual inspection. Metrology was then performed to assess the topography and mechanical wear of the telescopic bars, using a Talyrond 365 (Taylor Hobson, Leicester, UK) roundness measuring machine.

**Results:**

Plain radiographs showed evidence of a broken internal mechanism in 29% of retrieved rods. Single-side wear marks were found in 97% of retrieved rods. Material loss was found to significantly increase in rods with a damaged internal mechanism (*p* < 0.05) and rods with longer time in situ (*r* = 0.692, *p* < 0.05).

**Conclusions:**

We found an association between damage to the internal mechanism of the rods and (1) patterns of single-side longitudinal wear marks and (2) increased material loss. As the material loss was also found to increase over time of rod in situ, we emphasise the importance of early detection and revision of failed MAGEC rods in clinical practice.

## Background

Early-onset scoliosis (EOS) refers to a deformed spine with lateral curvature greater than 10 degrees in children under 10 years of age. In the UK, around 4 out of every 1000 children are affected by scoliosis, while EOS represents approximately 10% of all these cases [1]. Compared with scoliosis in other age groups, EOS children are at a stage of rapid growth and therefore subjected to a higher risk of curvature progression and secondary physical problems, such as restricted pulmonary development and neurological impairments. Clinical management includes observation, bracing and surgery. Distraction-based spinal rods are the most commonly used implants for surgical treatment, including traditional growing rods (TGRs) and MAGnetic Expansion Control (MAGEC) growing rods.

**Fig. 1:**
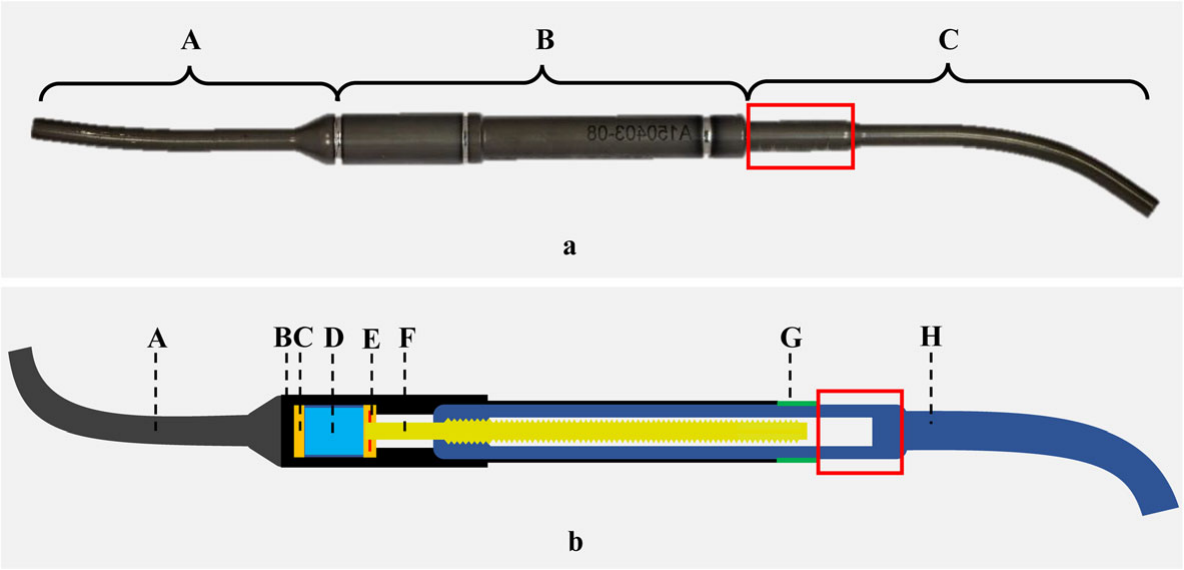
Photograph of a retrieved MAGEC rod (**a**) with the main components labelled as non-extending bar (*A*), actuator (*B*) and telescopic bar (*C*). Schematic of the mechanism of a retrieved MAGEC rod (**b**): From left to right: non-extending bar (*A, dark grey*), actuator case (*B, black*), bearings (*C, orange*), actuating magnet (*D, light blue*), locking pin (*E, red*), driving screw (*F, yellow*), sealing (*G, green*), telescopic bar (*H, dark blue*). The measuring region of this study was labelled (*box*)

The MAGEC system (NuVasive, Inc., USA) was approved in Europe in 2009 and recommended by the National Institute for Health and Care Excellence in 2014 as a treatment option for EOS children over 2 years old with indications of surgical intervention. A MAGEC rod consists of an internal actuating magnet, locking pin, threaded driving screw and bearings and external actuator case, sealing, non-extending bar and telescopic bar (Fig. 1). With the use of a magnetic drive lengthening mechanism, the MAGEC system avoids the repeated invasive lengthening operations, which are required for children with TGRs, while more frequent distractions are allowed to better mimic patients’ spinal growth. Primary outcomes of its use have been reported to be satisfactory in terms of deformity correction, incidence of implant-originated complications, mental burdens on children and overall cost [2–6]. However, complications and early revisions are common in patients receiving MAGEC rods, reported to be as high as 44.5 and 33%, respectively [7], of which the exact mechanism remains obscure.

Previous studies of retrieved MAGEC rods have suggested the relationship between the mechanical wear in retrieved rods and metallosis in surrounding tissue [8]. A recent retrieval study of 9 rods indicated that body fluid ingress and corrosive debris might account for the fractures of locking pins [9]. More recently, surface wear marks on the opposite side of both ends of the telescopic bar were suggested to result from ‘off-axis loading’, which referred to the axial deviation of rod lengthening and the impingements between telescopic bars and actuator cases due to the non-axial anatomy of human spine [10]. However, the role of mechanical wear in the failure of MAGEC rods remains elusive, while the cause of pin fractures is still not fully understood.

This study aimed to assess (1) the internal mechanism of retrieved MAGEC rods, (2) mechanical wear in the telescopic bars of retrieved rods and (3) the relationship between mechanical wear and the state of the internal mechanism.

## Methods

This was a retrieval study of 34 MAGEC rods (NuVasive, Inc., USA) explanted from 20 patients from multiple centres in the UK from 2014 to 2019. All rods underwent standard decontamination procedures using 10% formalin, upon receipt at our centre. There were no additional sterilisation methods applied, which were known to impact retrieval analysis findings. Six rods were in single construct and 28 rods were in dual construct. Patient information was collected for comparative purposes. The median (interquartile range (INQ)) age at implantation (years) was 10 (9–12). The time to explantation (months) was 34 (24.5–38.5). Eighteen rods were retrieved from unplanned revisions for progression of scoliosis, infection, anchor prominent or failure to distract, while 16 rods were from planned revisions for final fusion or maximum distraction reached.

### Plain radiographs

**Fig. 2:**
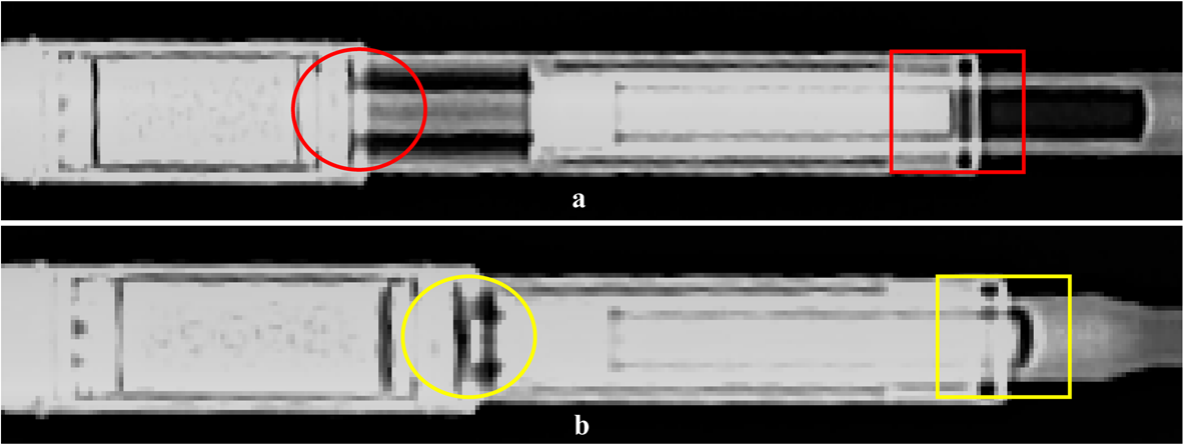
Plain radiographs of two MAGEC rods with intact (**a**) and damaged (**b**) internal mechanism. In the first rod (**a**), the connection of internal mechanism was intact (*circle*) and the internal driving screw was within the actuator case (*box*). In the second rod (**b**), the internal mechanism was disconnected (*circle*) and the internal driving screw was beyond the actuator case (*box*)

We hypothesised that analysing mechanical wear could help understand the failure of the internal mechanism in retrieved MAGEC rods. Using a medical X-ray device at 60 kV and 4 mA, the internal mechanism of all the rods was assessed by plain radiographs. According to a recent notice of MAGEC system announced by the manufacturers in June 2019 [11], the internal mechanism was deemed to be damaged when the internal components were disconnected or the internal driving screw was beyond the actuator case, both as a result of pin fractures (Fig. 2).

### Visual inspection

Macroscopic inspection was performed to visually assess surface mechanical wear in the telescopic bars of all the rods, using a digital single-lens reflex (Canon EOS 5D Mark II, Canon Inc., Japan).

### Surface mapping

**Fig. 3:**
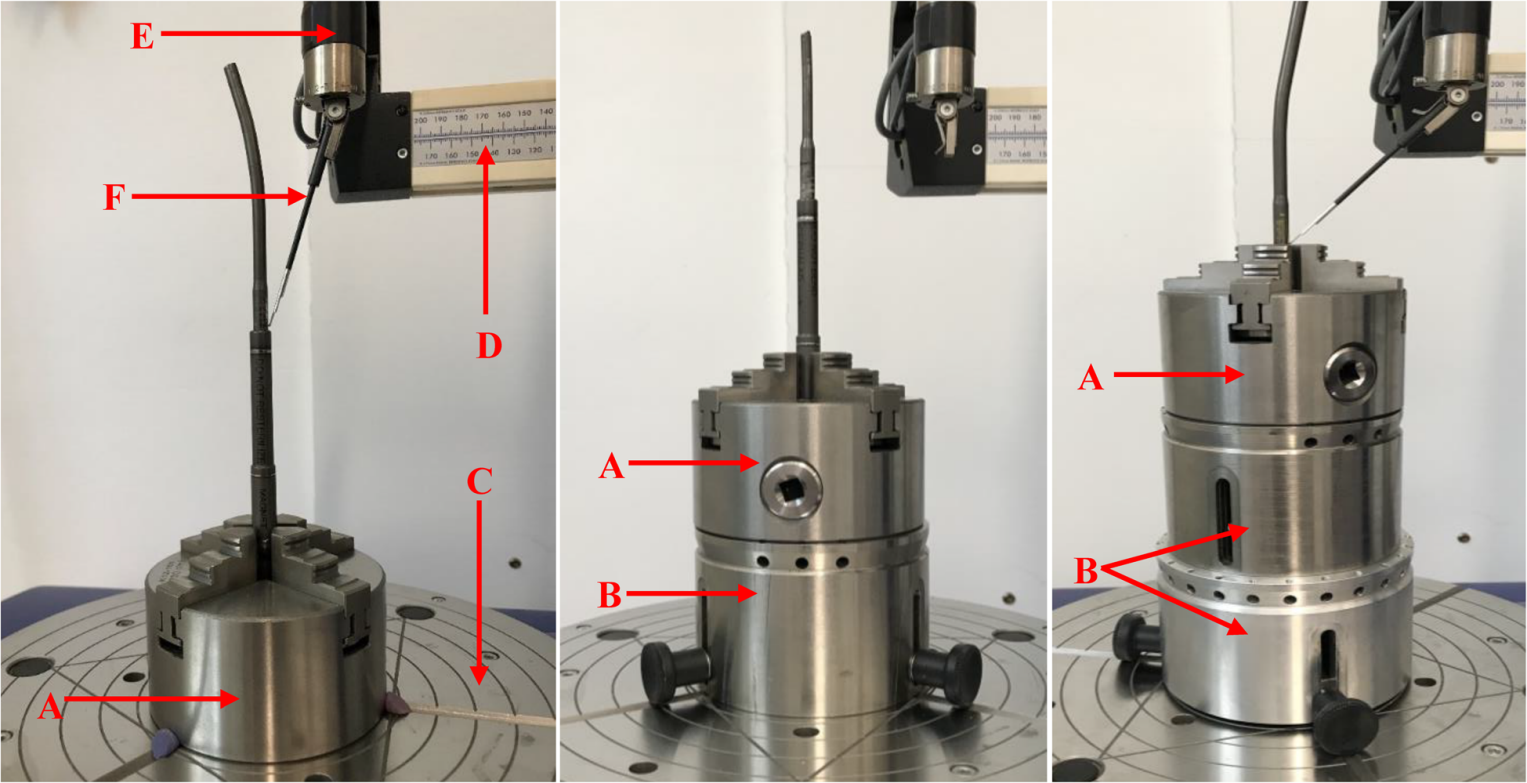
Photographs of three MAGEC rods fixed on the spindle of a Talyrond machine using a clamping fixer (**a**) and additional holders (**b**) with machine setting labelled as rotating air spindle (**c**), moving arm (**d**), measuring gauge (**e**) and diamond probe (**f**)

Three-dimensional surface mapping was used to assess the topography of mechanical wear in the proximal part of telescopic bars (Fig. 1) of all the rods, using a Talyrond 365 (Taylor Hobson, Leicester, UK) roundness measuring machine and a 5 μm diamond probe. The examined rod was firmly mounted in the centre of the spindle of the machine using a clamping fixer and additional holders (Fig. 3). The axial length (mm) and horizontal broadness (°) of visible wear were measured using the machine. After the alignment of the measuring region and the probe, serial traces were taken along the vertical axis of the rod at an interval of 10° (*Map 1*), starting from the bottom of the telescopic bar. With the use of TalyMap Gold 7.1 profiler software, a surface contour map was obtained and then levelled relative to the unworn region [12]. The resulting map depicted the profile of surface wear using a colour scale.

### Quantitative assessment of material loss

**Fig. 4:**
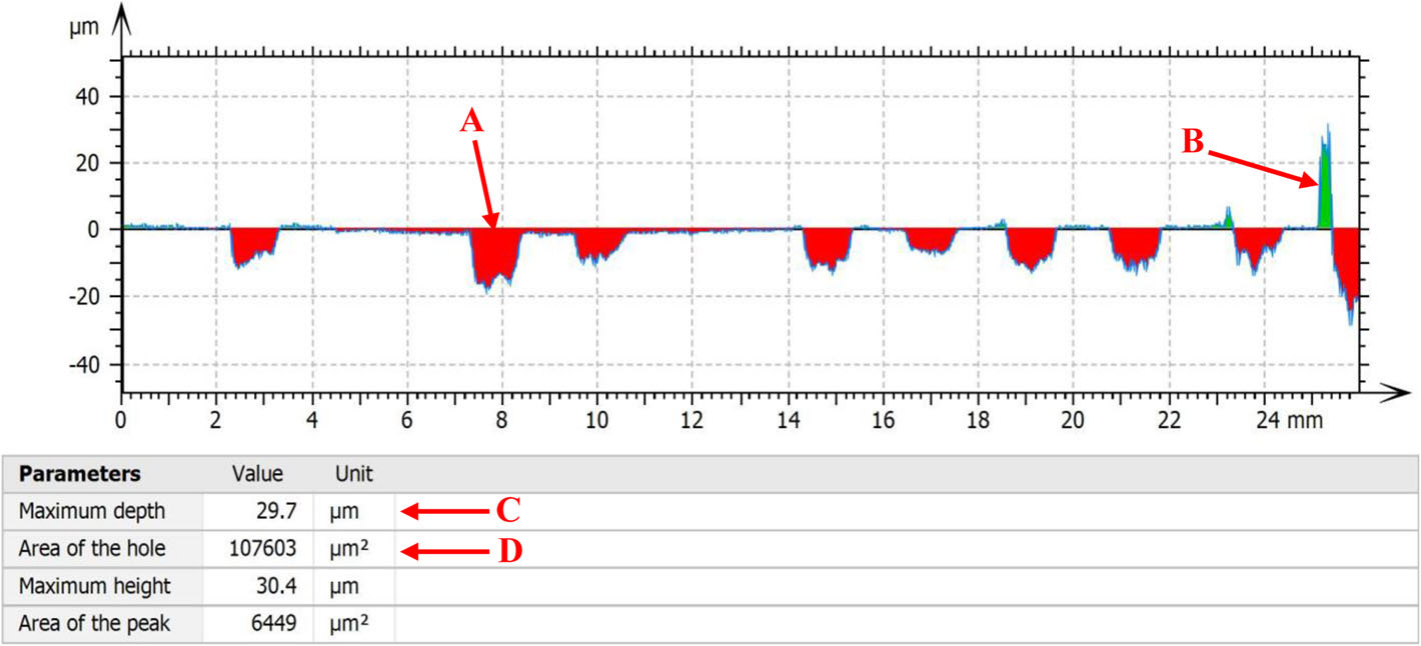
Profile of a trace extracted from the contour map of the telescopic region of a MAGEC rod. Surface wear was depicted as several valleys (**a***, red*), while surface debris was shown in green (**b***, green*) The maximum depth of these valleys (**c**) and the summed area of all the valleys (**d**) were calculated

Based on the contour map of visible wear, the magnitude of material loss from mechanical wear in the telescopic bars of all the rods was assessed by a further surface mapping in the most worn region with serial traces spaced at 1° (*Map 2*). After the extraction of trace profile from the levelled map, the area of wear in each trace was quantified with a measure of the maximum depth (μm) and summed area of all valleys (mm^2^) (Fig. 4) [13]. According to the value of maximum depth, five traces with the deepest wear were included for each rod in this study. The medians of the two values of five traces in each rod were recorded as wear depth (μm) and wear area (mm^2^), taken as a measure of material loss in each rod. Normalised wear area (mm^2^/mm) relative to measuring length (mm) was calculated for further comparative purposes.

### Repeatability and reproducibility

The intra-observer repeatability of surface mapping and material loss quantification were assessed with the Talyrond scanning and TalyMap calculations repeated by the same examiner in 6 and 15 rods, respectively. The inter-observer reproducibility of material loss quantification was verified with the TalyMap calculations repeated in 10 rods by a second examiner.

### Statistics

Statistical analysis was conducted using GraphPad Prism (version 8.0.1, US). Measurable results were presented as median (INQ). Differences were deemed to be statistically significant when two-tailed *p*-value was less than 0.05. Descriptive analysis was used to summarise all the results. Spearman correlation test was adopted to assess the relationship between material loss and patient demographics. Mann-Whitney test and Kruskal–Wallis test were used to compare material loss between two or more groups of rods, of which the results were shown as Box and Whisker plot. The repeatability and reproducibility were assessed using a Mann-Whitney test.

## Results

All the assessments were performed in all the retrieved rods with obtained results summarised in Table 1.

**Table 1:** Results of plain radiographs, visual inspection, surface mapping and material loss assessment

Case	Rod	Rod type	Lot number	Plain radiographs	Visual inspection (wear marks)	Map 1	Map 2	Wear depth (μm)	Wear area (mm^**2**^)	Normalised wear area (mm^**2**^/mm)
1	1	1.2	A130313–03	intact	circumferential	unmatched	matched	41.2	0.428	0.071
	2	1.2	A130523–01	intact	circumferential	unmatched	unmatched	1.9	0.008	0.001
2	3	1.2	A130130–02	intact	circumferential	matched	matched	95.8	2.757	0.084
3	4	1.2	120,314–002	unclear	circumferential	unmatched	unmatched	5.8	0.002	0.001
4	5	1.2	A140702–09	intact	circumferential	matched	matched	62.2	0.588	0.098
	6	1.2	A140605–01	dislocated thread	longitudinal	matched	matched	131.0	0.567	0.142
5	7	1.2	A141014–17	intact	circumferential	matched	matched	26.9	0.063	0.016
	8	1.2	A150220–08	intact	no	unmatched	unmatched	2.3	0.005	0.002
6	9	1.1	110,112–010-017	intact	circumferential	matched	matched	38.2	1.169	0.032
7	10	1.2	A130614–04	fractured pin	combined	matched	matched	103.0	2.918	0.224
	11	1.2	A130306–03	dislocated thread	combined	matched	matched	85.4	3.946	0.219
8	12	1.2	A130604–10	fractured pin	combined	unmatched	matched	129.0	12.873	0.348
9	13	1.2	A140127–09	intact	circumferential	unmatched	unmatched	12.2	0.060	0.002
	14	1.2	A130919–13	intact	circumferential	matched	matched	28.7	0.747	0.026
10	15	1.3	A150403–08	intact	circumferential	matched	matched	15.8	0.087	0.005
	16	1.3	A150717–14	intact	circumferential	matched	matched	60.7	2.574	0.135
11	17	1.3	A150728–01	intact	circumferential	matched	matched	25.6	0.077	0.013
	18	1.2	A140702–09	intact	circumferential	matched	matched	26.9	0.518	0.020
12	19	2.0	A150519–14-00	fractured pin	longitudinal	unmatched	matched	92.4	3.424	0.245
	20	2.0	A150519–12-00	fractured pin	longitudinal	unmatched	unmatched	48.9	1.252	0.125
13	21	1.2	A140604–05	intact	circumferential	matched	unmatched	89.0	4.675	0.195
	22	1.2	A140220–03	intact	circumferential	matched	matched	44.0	0.161	0.032
14	23	1.2	A131111–06	unclear	circumferential	unmatched	unmatched	10.2	0.004	0.002
	24	1.2	A140414–06	intact	circumferential	matched	matched	27.2	0.051	0.026
15	25	1.2	A140425–12	intact	circumferential	matched	matched	65.2	1.147	0.048
	26	1.2	A140127–09	intact	circumferential	unmatched	matched	31.4	0.168	0.013
16	27	1.2	A140301–05	intact	circumferential	matched	matched	52.7	1.423	0.119
	28	1.2	A140813–02	intact	circumferential	matched	matched	88.5	1.187	0.148
17	29	1.2	A140604–05	intact	circumferential	matched	matched	65.4	1.728	0.052
	30	1.2	A131111–06	intact	circumferential	matched	matched	15.8	0.367	0.015
18	31	1.2	A140127–09	fractured pin	longitudinal	matched	matched	16.7	0.099	0.008
19	32	1.2	A141222–01	fractured pin	circumferential	matched	matched	9.9	0.036	0.009
20	33	1.2	A140308–03	fractured pin	combined	matched	matched	168.0	10.356	0.414
	34	1.2	A140127–08	fractured pin	combined	matched	matched	99.4	4.043	0.225

### Plain radiographs

**Fig. 5:**
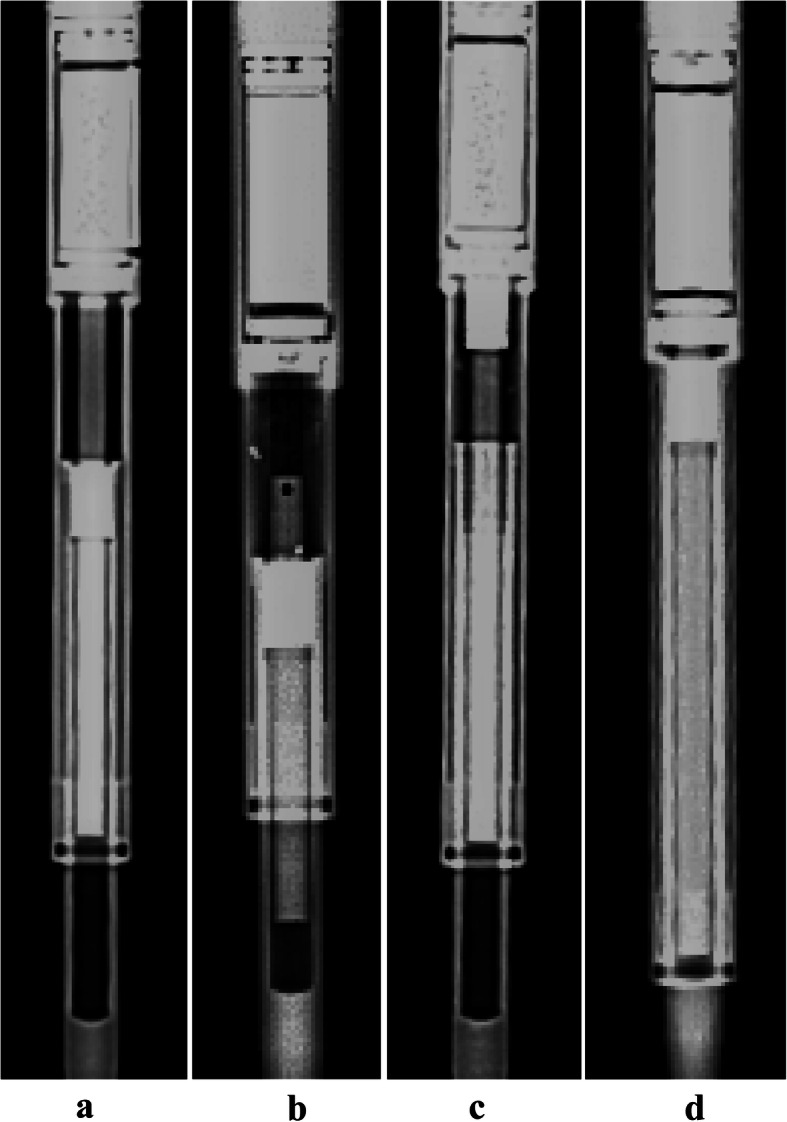
Plain radiographs of four MAGEC rods showing an intact internal mechanism (**a**), locking pin fracture (**b**), dislocated internal thread of the telescopic bar (**c**) and unsure status of internal mechanism (**d**)

An intact internal mechanism was observed in 22 rods (65%), while 10 rods (29%) showed evidence of a damaged internal mechanism (Fig. 5). Fractures of locking pins were detected in 8 rods (24%), while the internal thread mechanism of telescopic bars was dislocated in 2 rods (6%). The status of internal mechanism was unclear in 2 rods (6%).

### Visual inspection

**Fig. 6:**
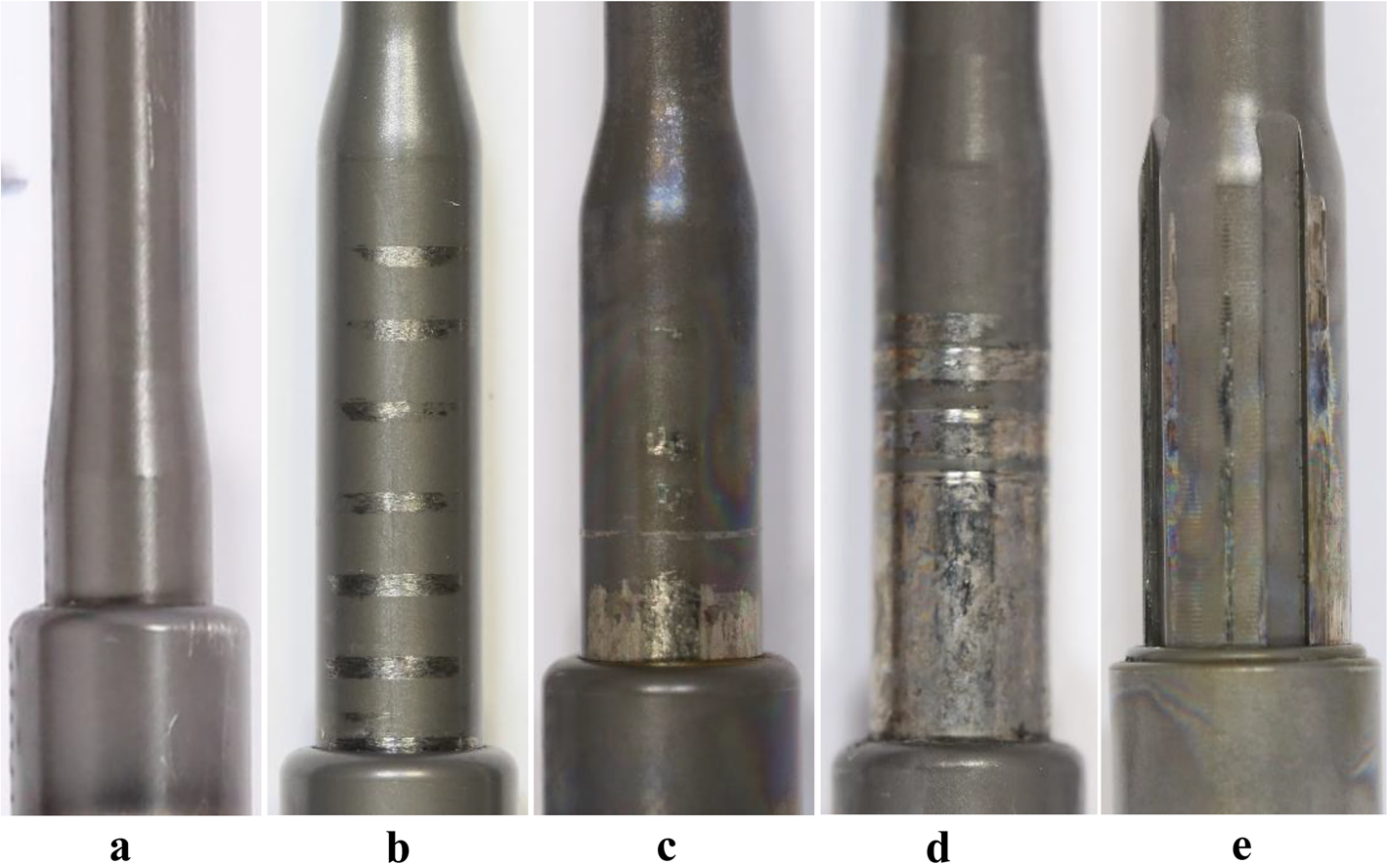
Photographs of the telescopic region of five MAGEC rods showing no wear mark (**a**), circumferential wear marks (**b**), longitudinal wear marks (**c**), combined wear marks (**d**) and a modified telescopic design (**e**)

Thirty-three rods (97%) showed evidence of wear marks on one side of the telescopic bars (Fig. 6). Circumferential wear marks were seen in 24 rods (71%), while longitudinal wear marks were noticed in 9 rods (27%), of which 5 rods were combined with circumferential wear marks. Two rods (rod 19, 20) showed modified design in the telescopic bars (Fig. 6e).

### Surface mapping

**Fig. 7:**
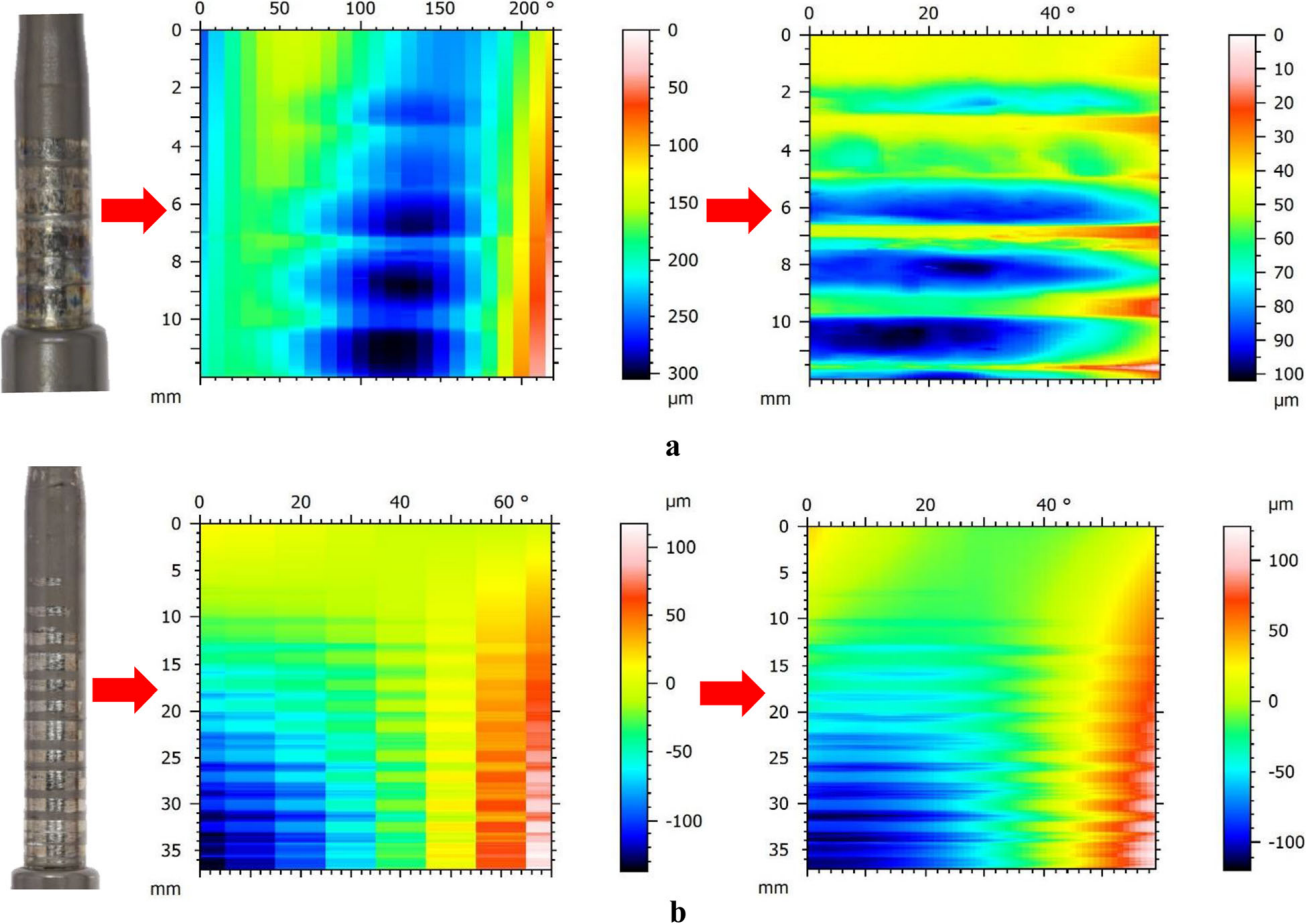
Photographs and surface contour maps of the telescopic region of two MAGEC rods. Each rod was mapped twice with serial traces taken at 10° and 1° interval in the visible worn and the most worn regions, respectively. The surface maps corresponded to the visible wear in the first rod (**a**), while the surface maps of the second rod failed to depict the actual wear profile (**b**)

The median (INQ) of the measuring length (mm) was 13 (5.8–25), while the median (INQ) number of traces was 12.5 (9–16.5). The resulting contour maps of 24 rods (71%) corresponded to the visible wear marks in the telescopic bars, while surface mapping in 10 rods (29%) failed to reflect the actual profile of surface wear (Fig. 7).

### Quantitative assessment of material loss

For surface mapping in the most worn region, the median (INQ) number of traces was 60 (42.3–60). The obtained contour maps of 27 rods (79%) corresponded to the visible wear marks (Fig. 7). As for the quantification of material loss, the medians (INQ) of wear depth (μm), wear area (mm^2^) and normalised wear area (mm^2^/mm) were 42.6 (16.5–88.6), 0.577 (0.074–2.619) and 0.040 (0.012–0.143), respectively. No significant difference was found in the material loss between single and dual rods (*p* > 0.05). The axial length of the measuring region was found to positively correlate with the wear depth (*r* = 0.38, *p* < 0.05), wear area (*r* = 0.63, *p* < 0.05) and normalised wear area (*r* = 0.35, *p* < 0.05).

**Fig. 8:**
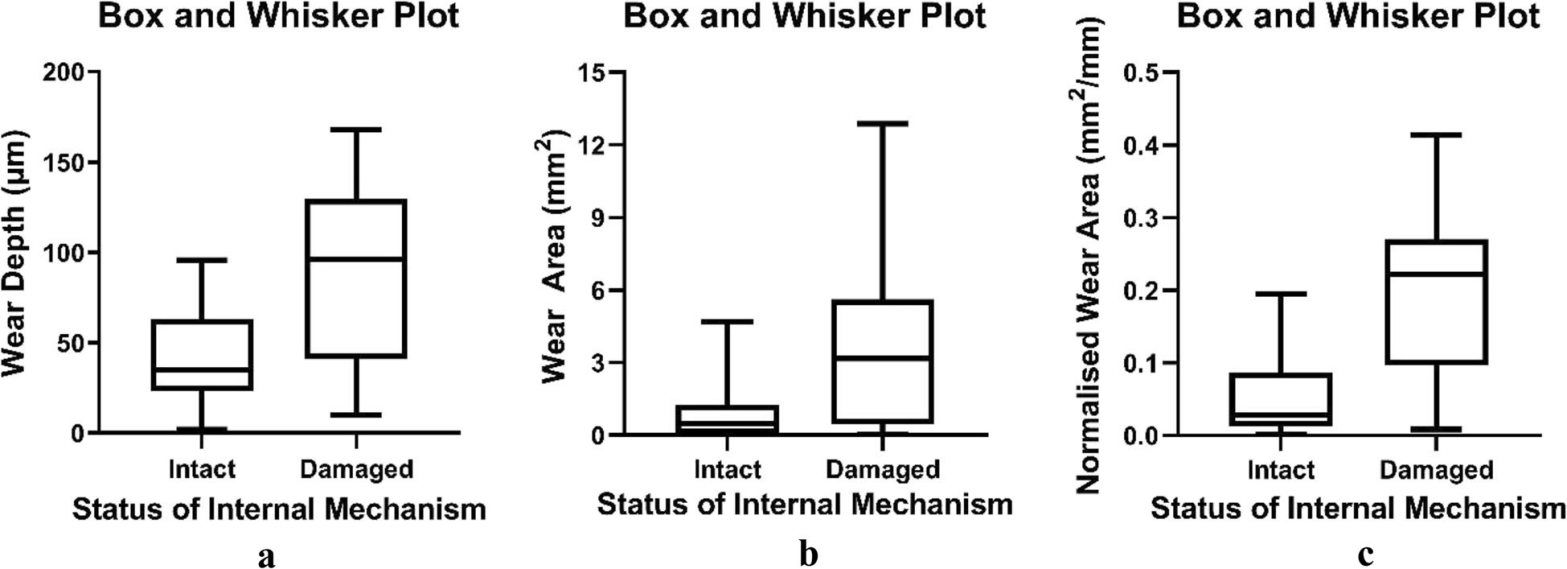
Box and Whiskers plot presenting the significant difference in wear depth (**a**), wear area (**b**) and normalised wear area (**c**) between retrieved MAGEC rods with intact and damaged internal mechanism (*p* < 0.05)

Mann-Whitney test found a significant difference in the three material loss values between rods with intact and damaged internal mechanism (*p* < 0.05) (Fig. 8). For the damaged group, the medians (INQ) of these three values were 95.9 (40.9–129.5), 3.171 (0.450–5.621) and 0.222 (0.096–0.270), higher than that of the intact group, i.e. 34.8 (23.2–63.0), 0.473 (0.074–1.246) and 0.029 (0.013–0.087). No significant difference was found in these three values between rods with different types of damages in the internal mechanism (*p* > 0.05).

Spearman correlation test found a significant and positive correlation between time to explantation and wear area (*r* = 0.433, *p* < 0.05), while a stronger correlation (r = 0.692, p < 0.05) was found between time to explantation and the mean wear area of dual constructs. No significant correlation was found in the wear depth and normalised wear area between the two variables (*p* > 0.05).

Kruskal-Wallis test showed a significant difference in the wear area between different pre-operative diagnosis groups (*p* < 0.05), although no significant difference was found in wear depth and normalised wear area between these groups (*p* > 0.05). By comparing material loss between any two diagnosis groups, the idiopathic scoliosis group was found to have significantly higher medians (INQ) of the three material loss values (*p* < 0.05), i.e. 50.8 (26.6–71.2), 1.167 (0.166–1.939) and 0.050 (0.014–0.139), compared with 13.5 (9.9–24.6), 0.043 (0.012–0.087) and 0.009 (0.004–0.021) for the syndromic scoliosis group.

The rods of planned revisions were found with increased wear area (mm^2^), i.e. 1.158 (0.162–2.878), compared with 0.473 (0.056–1.711) for the rods of unplanned revisions, although the difference was not significant (*p* > 0.05). No significant difference was found in the material loss between different reasons for unplanned revision (*p* > 0.05). No significant relationship was found between the three material loss values and the gender and age at implantation of patients (*p* > 0.05).

### Repeatability and reproducibility

No significant difference was found in the three material loss values between two intra-observer repeats of Talyrond scanning and TalyMap calculations as well as the two inter-observer repeats of TalyMap calculations (*p* > 0.05).

## Discussion

The MAGEC system has been increasingly used for treating EOS patients due to its non-invasive lengthening. However, unplanned revisions of MAGEC rods due to a failure of the internal mechanism remains a concern of its use [14, 15], of which the mechanism is still unclear. This is one of the first studies to analyse retrieved MAGEC rods using high-precision metrological techniques, which has been widely used in retrieval analysis of hip implants [16, 17].

In the current study, almost one third of the rods revealed evidence of a damaged internal mechanism. Among the rods manufactured before March 26, 2015 and those manufactured on or after March 26, 2015, the rates of pin fractures were found to be 6 out of 29 (21%) and 2 out of 5 (40%), respectively. It had been reported by the manufacturer in a field safety notice that the rate of fracture before and after this date was 5 and 0%, respectively [11]. The differences in our study are likely due to the different denominator used to calculate percentage, due to the relatively smaller sample size in our study. Intensive monitoring of rod integrity is of great importance in clinical practice however, especially for patients with older batches of rods.

**Fig. 9:**
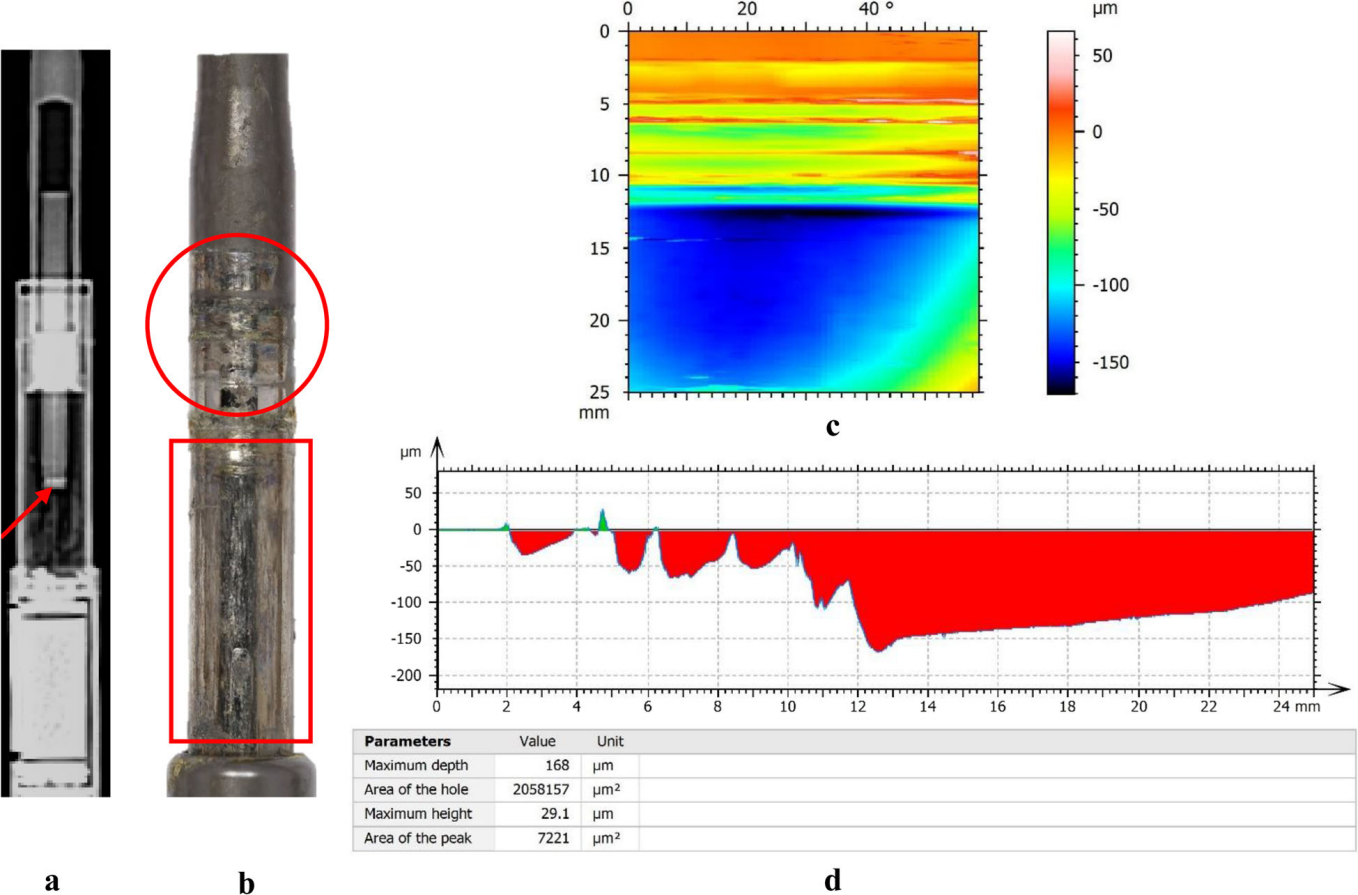
Plain radiograph (**a**), photograph (**b**), surface contour map (**c**) and trace profile (**d**) of a retrieved MAGEC rod with a pin fracture. We suggest that ‘off-axis loading’ accounts for the initial spaced wear marks (*circle*) and the pin fracture (*arrow*) and the subsequent uncontrolled lengthening of the rod contributes to the longitudinal wear marks (*box*) and further massive material loss

Single-side wear marks were seen in the telescopic bars of almost all the rods in our study, indicative of the effect of ‘off-axis loading’ proposed by a previous study [10]. We found that rods with a damaged internal mechanism showed a prominent wear pattern [9]. Specifically, except for one rod (rod 32) that likely failed to distract after its pin fracture, all the other nine rods with damaged internal mechanism showed longitudinal wear marks, of which five rods showed a mixture of spaced circumferential and consecutive longitudinal wear marks. Coupled with the significant increase of material loss detected in retrieved rods with a damaged internal mechanism, we suggest that ‘off-axis loading’ accounts for the initial spaced wear marks and the breakage of the internal mechanism, while the subsequent uncontrolled lengthening of telescopic bars driven by patients’ growing spine contributes to the longitudinal wear marks and further increased material loss (Fig. 9).

We found that increased wear area was associated with increased time in situ of the rod, which however had no significant effect on the wear depth and normalised wear area. Hence, we suggest that the amount of material loss from implanted MAGEC rods accumulated with time, which also explained the grossly increased wear area in the rods of planned revisions. As the current study may be underpowered, future work should incorporate more rods for analysis. Considering the structural intactness in the rods of most planned revisions, increased in-situ time is suggested to be another possible contributing factor to the increased material loss from implanted rods, which do not necessarily present any structural damages. The increased material loss detected in the current study also provides evidence for the regional inflammatory reaction and increased serum ion levels reported in patients receiving the rods [8, 18]. Long-term follow-ups are needed to understand the impact of increased material loss on patients’ health. Therefore, local and systematic clinical care is still warranted in cases with intact rods in situ.

Of note, we found that the rods with more distraction gain presented deeper wear and increased material loss per unit. One explanation was that the bending moment on the telescopic bar was increased as the rods extended, leading to severer ‘off-axis loading’ and material loss. This may explain the frequent ‘diminishing returns’ or slippage during distraction observed in rods reaching half of the maximum extendable length [19, 20] and the angulated telescopic bars noticed on radiographs [21]. Besides, it has been suggested that a single rod construct was subjected to a higher bending moment and therefore at a higher risk of breakage [22]. However, our study found no difference in material loss between single and dual rods, which was likely due to the relatively small sample size.

Out of all the unplanned revisions, only 6 rods (rod 6, 12, 31, 32, 33, 34) were noted with dislocated thread or pin fractures, while the causes of unplanned reoperations showed no significant effect on the material loss in this study. Further studies are necessary to correlate clinical reasons for unplanned reoperations with the structural integrity and material loss of MAGEC rods.

Out of all the planned revisions, 4 rods (rod 10, 11, 19, 20) were found with structural damages. Although previous studies have suggested that ‘clunking’ or ‘stalling’ may occur during rod distractions due to the failure of internal mechanism [23], the clinical records of these rods did not show any evidence of structural damages in distraction clinics prior to the planned revisions. We suggest that the evidence of internal damages from retrieval analysis does not necessarily correspond to the functional changes of the rod in the clinic. We emphasise the importance of considering surgeon, implant and patient factors when examining retrieved rods. Moreover, it is of great interest to correlate the occurrence of clunking during distractions with the material loss of the rods in future studies.

This study helps provide one explanation for the failure of internal mechanism of MAGEC rods, but the role of other failure mechanisms of MAGEC rods is yet to be explained. As the first attempt to apply surface mapping in MAGEC rod assessment, the accuracy of our mapping protocol was verified with most of the contour maps corresponding to the actual wear profile in retrieved rods. Nevertheless, further improvements are necessary to increase the accuracy of surface mapping in MAGEC rods, as surface mapping of a higher resolution was only achieved in a relatively narrower region in our study.

## Conclusions

This study is one of the first to apply metrological techniques to investigate the failure of retrieved MAGEC rods. We found that ‘off-axis loading’ was one of the causes of the damage of internal mechanism in retrieved rods, indicated by the one-side longitudinal wear marks and increased material loss in the telescopic bar of these rods. Since the material loss was also found to accumulate over time of rod in situ, we emphasise the importance of early detection and revision of failed MAGEC rods in clinical practice.

## Data Availability

The datasets used during the current study are available from the corresponding author on reasonable request.

## Abbreviations

EOS: Early-onset scoliosis
TGRs: Traditional growing rods
MAGEC: MAGnetic Expansion Control
INQ: Interquartile range
